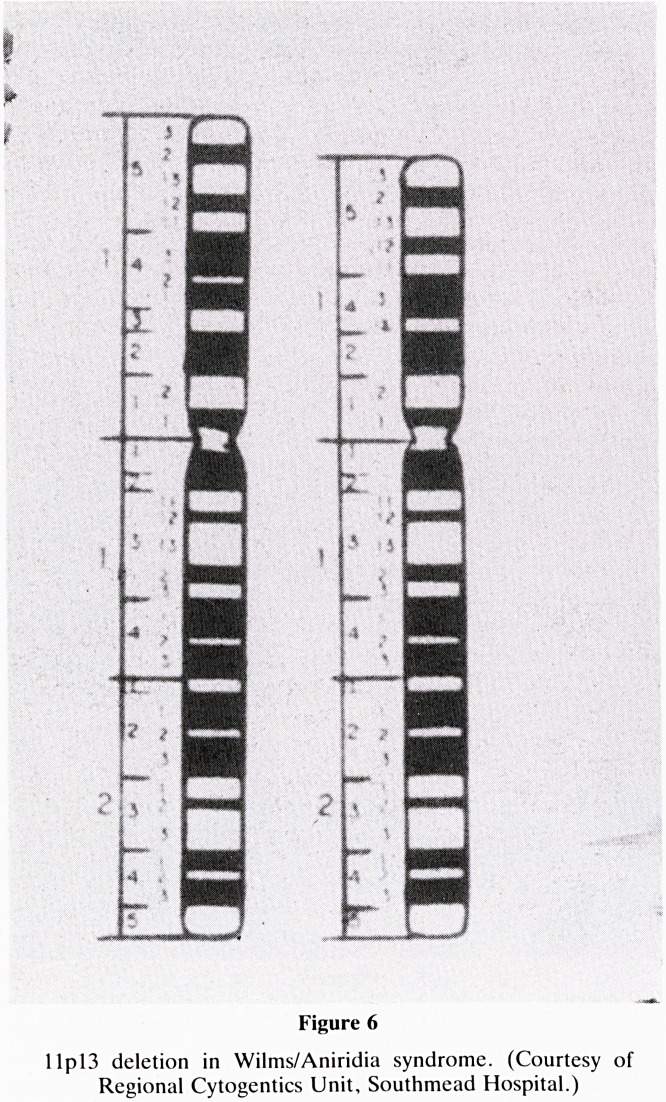# Malformation, Mutation and Malignancy

**Published:** 1989-02

**Authors:** Martin Mott

**Affiliations:** Reader in Paediatric Oncology, Institute of Child Health, Bristol Children's Hospital


### Mutation, Malformation and Malignancy
### The 1987 Long Fox Lecture

**Martin Mott, BSc, MB, ChB, FRCP, DCH**
Reader in Paediatric Oncology, Institute of Child Health, Bristol Children's Hospital

This lecture honours the memory of Edward Long Fox, a major figure in the proud tradition of Bristol medicine. His grandfather, Edward Long Fox senior, was a physican at the Infirmary as was his Uncle also; clearly there was a strong family tradition for him to live up to.

Edward Long Fox junior was born in 1832, and educated at Bath and Shrewsbury before going up to Balliol College, Oxford. He commenced his medical studies in Edinburgh, but quickly moved to London. It is particularly interesting to a paediatrician that in 1855 he spent a period at Great Ormond Street Hospital, in its very early days. The Hospital for Sick Children was founded in 1851 by Charles West. It was the first Children's Hospital in the United Kingdom and the only one to pre-date our own Children's Hospital in Bristol. In a book for parents published by the hospital at the time (1), we learn that more than 40% of the deaths in London were of children less than ten years old and yet only 1% of hospital beds were available for that age group. Living conditions were grim as we know from much of the literature of the day, and the experience of working under the leadership of Charles West must have opened Long Fox's eyes to the impact of the environment early in his career.

He graduated in 1857 and was appointed as Physician at Bristol Royal Infirmary where he stayed for the next twenty years. His lectures on medicine and pathological anatomy at the Bristol Medical School were very popular with the students of the day, and he quickly established himself as one of the leading lights on the Bristol medical scene. He had a very successful practice in Clifton, and was much loved by his patients and respected by his colleagues, being elected President of the Bristol Medico-Chirurgical Society in 1881. He was well known outside Bristol and was influential in the Royal College of Physicians, where he became a Fellow in 1870 and gave the Bradshaw Lecture in 1882. He became a member of the Council of the Royal College of Physicians in 1888 and in 1894 he was elected President of the British Medical Association at its Annual Meeting in Bristol. His presidential address on 'The Medical Man and the State' (2), attests to his clearsighted understanding of the link between disease and the environment . . .'. Our aim is . . . that poverty may be mitigated by more healthy surroundings and that sickness may be diminished by the education of the nation on the wiser laws of health, by increased temperance and by a knowedge of the common facts of physiology from an early age'.

He died in 1902, much mourned by the people of Bristol.

### THE VICTORIAN ERA

Edward Long Fox was a man of his time, but he was also a man of insight and vision. His Obituary tells us both of his respect for the past and of his enthusiasm for the future. We can form an impression of the people who would have most influenced him by noting the giants of that era who died around the time that he qualified. The names and reputations of Richard Bright, Thomas Addison and Thomas Hodgkin are familiar even today. At the same time, three great books were published, which must have had a powerful influence upon him: Virchow's great treatise on Cellular Pathology; Charles Darwin's momentous work on the Origin of Species, in which he explained the causal association between individual variation and the process of evolution by natural selection — the survival of the fittest; and Charles Dicken's Great Expectations, which along with his other works provides us with such a memorable portrait of the way things were in 19th century Britain.

A closer understanding of his world comes from looking at some of his contemporaries: we see among them in particular many of the founders of the new sciences of Microbiology and Genetics. To the paediatrician interested in growth and developoment, and in the interplay between the genetic endowment we inherit from our parents and the environment we live in and likewise inherit, it is apparent that the effects of the environment were paramount in the 19th century, as can be seen by looking at the causes of death among children at that time (Fig 1).

The infectious diseases have largely been conquered in our society due to the pioneering efforts of the contemporaries of Edward Long Fox, though we should remember to our shame that they still exact a fearful toll among children worldwide. Last year fourteen million children under the age of five years died, only 2% of those deaths being in the industrialised countries. The causes of death in the vast majority of these were infectious diseases which we have had the capacity to eradicate for decades, if only we had the political and social determination to do so. A tiny proportion of the budget we spend on the unnecessary luxuries that damage our health and wellbeing, or on the weapons of war and mass destruction which we continue to accumulate, would be more than enough to feed the starving and immunise and protect these children from infectious diseases. Their deaths are an indictment of the continued greed and selfishness of our so called civilised society.

### CRITICAL DEVELOPMENTS

The major developments in the understanding of the infectious diseases that took place in the latter half of the 19th century paved the way for truly dramatic reductions in morbidity and mortality in the early half of the 20th. In the latter part of the 20th century, an equivalent revolution in understanding is taking place in the field of molecular biology and I hope to show you how this new understanding is likely to revolutionise medical practice in the 21st century.

**Figure 1**

Charles Darwin's book on the Origin of Species serves as a paradigm for the dramatic changes in the thought processes of the scientists and physicians of his day. A century later, an equivalent landmark publication was that appearing in 1953 by James Watson and Francis Crick on the nature of the structure of DNA, the so called double helix (3). This can be taken as a starting point for the new revolution of molecular biology. The ensuing thirty years has seen a phenomenal growth in our knowledge and understanding of molecular biology including the establishment of the complete genetic code in 1966, detailing the specific triplets of purines and pyrimidines which code for the individual amino acids that go to make up any protein chain. Just five years ago it was shown that a single base change in a gene concerned with normal growth and development is sufficient to initiate cancer. (4) Molecular biology makes it possible to look at the processes of growth and development at an entirely new level. It took the genius of Rudolph Virchow to relate the gross morphologic changes observed in tissues undergoing abnormal growth and development to aberrant processes going on at a cellular level. Now we are able to go a stage further to relate those changes to events at a molecular level in the very heart of the cell.

### THE NEW TECHNOLOGY

Since the genetic code was established, we have developed a much clearer picture of how genes work as blueprints for the formation of proteins in the cell. The blueprint in DNA (genes) is transcribed into RNA and inessential parts of this message are then spliced out before it is translated into amino-acids to form a protein. It is now possible to examine the precise sequence of bases in DNA and RNA, thanks to the discovery of enzymes called Restriction Endonucleases which recognise and cut highly specific sequences in DNA, always at precisely the same point. The cut fragments of DNA, are electrophoresed on a gel and the different sized restriction fragments migrate at different rates. A piece of complementary DNA can then be used as a radioactive probe

**Figure 2**

Loss of heterozygosity for gene probes at 11p13 in a series of patients with Wilms tumour when comparing their normal tissues (N) with their tumour (W). (Courtesy of Andrew Shaw.)

to seek out the specific sequence under investigation on an autoradiograph. (Fig 2) DNA 'restriction fragments' can be inserted into plasmids and multiplied in bacterial culture (cloned) to any quantity desired. The precise sequence of a DNA fragment can be read by labelling the individual bases of which it is made. Once the sequence of bases is established, the genetic code is used to work out the sequence of the messenger RNA and of the protein for which that gene is a blueprint.

### DIFFERENTIATION

As cells grow and divide, some of them begin to take on different characteristics according to which particular genes in their reportoire are switched on to be active and which are switched off. The end result is a composite of several different types of tissue; although all the cells of an organism contain the same genetic material, the genes that they express and therefore their structure and function differ markedly from one set to another. Some genes are expressed only at certain times and are switched off at others, for example the genes which code for haemoglobins. While the foetus is oxygenated through the placenta, the requirements for oxygen carriage are different from those in the newborn baby, who obtains oxygen from the lungs. The genes which code for foetal haemoglobin are turned off as the foetus matures, and those that code for adult haemoglobin are switched on, so that the infant is prepared for the move from intra-uterine to extra-uterine life.

### PROLIFERATION OR DIFFERENTIATION

Cells have the capacity either to grow and multiply, or to stop dividing and develop specialised functions in different tissues; we have therefore to postulate at least two pathways along which any cell can travel. The first permits a continued process of self renewal, whereby a stem cell divides into two identical daughter cells which can each multiply or proliferate indefinitely. The second determines that the cell starts to develop along specialised lines; as it differentiates, so it progressively loses the capacity to proliferate, until eventually cells which are fully differentiated are no longer capable of proliferation. Our task is to understand how entry to these pathways is controlled and to discover what the messages are that allow cells to communicate with their neighbours and to coordinate their activities. When the messages between cells go wrong, then growth and development become disordered and the resulting malfunctions may be categorised as for example, malformations or malignancies.

### COMMUNICATION

How then do cells talk to each other? There are many possibilities but three examples of inter-cellular communication will suffice as an indication of the complexities involved.

The first (autocrine) is where the cell's genes programme it to make messages which react with receptors on its' own surface, resulting in a stimulus or feedback loop to the original cell. The second (paracrine) is where the cell produces a message which diffuses to its next door neighbours whose receptors are stimulated into appropriate action according to the nature of the message. The implications of a paracrine mechanism are substantial. Individual cells have their behaviour affected by the good or bad company they keep: a 'bad' cell whose behaviour is out of place may actually cause significant pathology to develop in its neighbours, especially if they are immature and of an impressionable age!

The third example (endocrine) is where cells secrete messages which are carried in the blood stream to distant parts of the body, to scattered 'target' cells that have specific receptors programmed for that particular message.

In each example, after a message has arrived at the cell membrane it has to be translated into action within the internal constituents of the cell. The cell membrane is a complex structure open to numerous possibilities as a transmitter. The basic essentials of the process can be depicted as follows (5). The external signal is taken up by specific receptor molecules in the outer border of the cell membrane and the signal is then transmitted through a series of steps to the intra-cellular compartment. There are at least two major pathways for this that have been identified and worked out in some detail. In both, the external signal is taken up by a receptor, transduced, amplified and passed to a second messenger in the intra-cellular compartment, which in turn acts on an internal receptor to stimulate the desired cellular response. Careful study of the various components of this signal pathway has revealed a whole series of individual proteins which turn out to be virtually identical to a group of proteins already investigated intensively in an entirely different context.

### GROWTH FACTORS AND 'ONCOGENES'

The amino acid sequence of these proteins is that which was predicted from the DNA sequence of genes that have been studied by tumour virologists for several decades. For example, a well characterised growth factor known as platelet derived growth factor (PDGF) has an amino acid sequence which corresponds to the genetic code contained in the simian sarcoma virus—the V-sis oncogene (6). The epidermal growth factor (EGF) receptor is a protein (a tyrosine kinase) whose amino acid constitution is known from the genetic code for the avian erythroblastosis virus (V-Erb B) (7). The Abelson Murine Leukaemia virus (V-abl) the Kirsten and Harvey Murine sarcoma viruses (V-Ki-ras and V-Ha-ras), the avian myelocytomatosis virus (V-myc) and avian myeloblastosis virus (V-myb) are just some of the forty other examples now known (8). The whole story, admittedly over simplified, can be summarised as follows: there is a class of viruses called retroviruses which have a habit of picking up genes from the host cell in which they multiply, and of carrying those genes with them when they go on to infect other cells. If the gene which is picked up happens to be the blueprint for one of the proteins concerned with growth and development, and if, as a result of being hijacked that gene and its protein are altered in some way, then the message to the subsequently infected cells may make their later growth and development abnormal.

Five years ago to the day, (5/11/82) careful and elegant studies of one of the oncogenes known to be involved in the development of a human bladder carcinoma were reported (4) They showed that the only change in this gene was a single point mutation of one base, a change from guanosine to thymidine. This resulted in a change in the triplet code so that the twelfth amino acid residue of the protein derived from the gene changed from glycine to valine. This single amino acid substitution was sufficient to result in the induction of cancer.

What other ways might normal genes involved in the process of growth and development be activated to become oncogenes (genes causing cancer)? Apart from a single point mutation, a second well documented instance is where the number of copies of the gene is amplified to many times the original number (9). A third way is by the carriage of the gene to a different part of the genome where it is integrated somewhere without its usual control genes beside it. The result of such a gene translocation may likewise be cancer.

### CHROMOSOMES AND MUTATIONS

The potential importance of chromosones was beginning to be appreciated at the turn of the century when the science of genetics was in its infancy. Edward Long Fox may well have been aware of some of the early work in this field and one wonders whether he may have seen the beautiful pictures of mitoses published by Van Benenden in 1883. It is more doubtful if he was aware of the work of Theodore Boveri, who performed a series of crucial experiments in the early part of the present century which culminated in the publication in 1914 of an hypothesis which was at least 50 years ahead of its time (11). It is only recently that all of the components of this hypothesis have come to be accepted. Boveri suggested that malignant cells arise from normal cells, and that they commence from a single cell which becomes cancerous, — that is, they are of clonal origin. He suggested that all malignant tumours would have an abnormal content of chromatin, the consequence of this abnormality being a capacity for unlimited proliferation. He suggested that processes which led to an abnormal chromatin constitution would predispose to the formation of tumours: examples he suggested were an abnormally heavy proliferation of cells, the ageing of cells, exposure to X rays or chemicals, and a genetic predisposition to malignancy. Just how right he was will become clear as we look at some of the discoveries of recent times.

Darwin's theory of evolution by natural selection predicted that individuals who develop features which are advantageous to their survival will tend to pass on those characteristics to future generations, whereas variations (mutations) which are disadvantageous tend to die out. It is important to understand that exactly the same concept is equally useful in explaining why, at a cellular level, some groups of cells may be more successful than others.

A Mutation is a change in the structure of the genetic material — that is in the DNA, or base sequences in the genes — which is inherited. Thus, if mutation occurs in the germ cells, it will be inherited in the next generation, whereas if it occurs in a somatic cell, it will be present only in the progeny of that cell. Any change in gene structure may alter gene function and thereby the structure and function of that cell and also the surrounding tissues with which the cell communicates. There are several different types of mutation, the simplest of which is a point mutation, where a single base is changed, thus altering the amino acid sequence of the protein for which that gene is a blueprint. A much larger mutation may be caused by deletion, duplication or translocation of a whole segment of a chromosome containing many genes, and this is likely to result in more profound alteration of that cell's qualities. Another type of mutation is aneuploidy or alteration in the number of chromosomes in the cell, usually due to the absence of a chromosome (Monosomy) or to the presence of an extra chromosome (Trisomy). Polyploidy defines a situation where a cell may have three or four sets of chromosomes instead of two.

### MUTATION, MALFORMATION AND MALIGNANCY

It has been known for a long time that certain chromosome anomalies predispose affected children to develop cancer. Perhaps the best known example is Down Syndrome or trisomy of chromosome 21, where children with multiple malformations are also much more likely than normal to develop acute leukaemia (12). We now know that chromosome 21 is important in a large number of leukaemias, not just those occurring in association with Down Syndrome.

A predisposition to cancer is inherited in a number of rare autosomal dominant conditions. For example, there are a number of malformations known to be associated in Gorlin's syndrome which often presents with the development of odontogenic keratocysts in the jaw. The father of our only patient developed a number of naevoid basal cell carcinomas in his thirties and his daughter developed cysts in her jaw in just the same way as had her father. She then developed an oropharyngeal rhabdomyosarcoma and was the second patient recognised to have this syndrome who has also been recorded to develop this particular rare cancer of childhood (13), although other childhood cancers such as Medulloblastoma have been more frequently reported. She was successfully treated for that tumour but has recently developed a crop of naevoid basal cell carcinomata at the back of her neck in the exit portal from the radiation beam used to treat her rhabdomyosarcoma.

There is a much broader range of autosomal recessive disorders in which childhood neoplasia has been observed. Typical among these are Fanconi's anaemia, Ataxia-Telangiectasia, Xeroderma-pigmentosum and Bloom's Syndrome. Apart from the fact that they share as features a variety of congenital malformations associated with a predisposition to develop cancer, there is an interesting common feature in a number of these syndromes, namely a basic defect in the ability to repair DNA, and this is almost certainly related to the predisposition to develop cancer (14). As in all autosomal recessive disorders, the frequency of heterozygotes in the community is much higher than the frequency of homozygotes who develop the full blown syndrome. This brings us to a fundamental question; what is the advantage that these heterozygotes have which enables the gene to reach this frequency in the population, despite the severity of the disease in the homozygous condition? Present molecular biology techniques should enable us to answer this question for many disorders in the next decade. The knowledge gained is likely to have a major impact on our understanding of many diseases and on our ability to treat them.

Sex linked recessive disorders are typified by a group of immunodeficiency diseases in which there is a marked predisposition to develop lymphoid malignancies (15).

There is, therefore, a strong association between malformations and malignancies. The associations of aniridia, (absence of the iris) with Wilms' tumour, and of hemihypertrophy, (where one side of the body is larger than the other), with tumours such as Wilms' tumour. (Nephroblastoma) Hepatoblastoma, and adrenocortical carcinoma merit discussion in more detail, as does the Beckwith–Wiedemann Syndrome, but the list of such associations is too long to allow more than a few examples. Another important related property of some embryonal tumours and of neonatal tumours in particular is their propensity for spontaneous regression (16). There appears to be some capacity in tumours occurring in early infancy to switch back from a proliferative mode to a differentiative mode, simulating what happens to cells in the normal fetus as it passes from the need for rapid multiplication of cells to a need for groups of those cells to undergo differentiation.

### ANTIONCOGENES

R. P. is a patient who inherited the familial form of retinoblastoma from her father. Becasue of the known pattern of inheritance, her eyes were examined soon after birth and her multifocal retinoblastoma was dealt with by radiation therapy. At the age of 4 she re-presented with a swelling in her cheek, which on biopsy was shown to be a malignant sarcoma, occurring on the edge of the radiation field. She subsequently went on to develop a third tumour, a pinealoblastoma, an association which is well documented (17). Patients who have this inherited predisposition tend to develop bilateral retinoblastoma in early infancy: they tend to develop secondary sarcomas in irradiated tissues and worldwide more than a dozen such patients have now been reported to develop pinealoblastoma, in what is effectively a third 'eye'. This constellation of findings is clearly related to the abnormality that causes the retinoblastoma in the first place. Such patients are in addition subject to develop other

**Figure 3**

Hemihypertrophy and hepatoblastoma in an infant with the Beckwith–Wiedmann Syndrome.

malignancies and if they survive, may well develop osteosarcoma of the long bones at a later age. The genetic abnormality that causes this syndrome has been known for some time to involve a deletion on the long arm of chromosome 13 (13Q—) and cytogenetic analysis of the cells of such patients regularly and reproducibly shows this disorder (18). Note that here we are talking of loss of genes as a cause for cancer, a subject to which we will now turn.

The Beckwith–Wiedemann Syndrome is otherwise known as the EMG syndrome for exomphalos, macroglossia, and gigantism, some of the cardinal features at presentation. There is a whole constellation of abnormalities described in this syndrome but the one that interests us in particular is the predisposition to cancer of a variety of types, all being primitive embryonal neoplasms, though the commonest is nephroblastoma. We have for example seen infants with this syndrome present with a huge abdominal mass due to a hepatoblastoma associated with hemihypertrophy (Fig 3). One recent patient on investigation of his cytogenetics showed trisomy for the short arm of chromosome 11, an abnormality that has been described previously in this syndrome (19). Both his father and paternal grandmother have a balanced translocation with a reciprocal interchange of chromosomal material between chromosomes 5 and 11. In his case, however, he has inherited this material in addition to two normal chromosome 11's and so is trisomic for this

**Figure 4**

Trisomy p11. (Courtesy of Regional Cytogenetics Unit, Southmead Hospital.)

**Figure 5**

Aniridia in a patient who developed Wilms' tumour.

portion (Fig 4). This could be an important clue concerning the aetiology of the syndrome and is of particular interest with regard to the aetiology of the predisposition to cancer. Unfortunately, cytogentics even with banding does not take us down to a level of resolution that will enable us to investigate single genes. For example, the gene for beta Globin is known to be on the short arm of chromosome 11 in band p12; but we know that that band alone contains over 5 million bases (5000 kb). The cluster of genes of the globin family are contained in a small 60 kilobase segment of that area, and the beta globin gene itself consists of some 1.6 kilobases. A real understanding of the genetic causes of cancer therefore requires the fine resolution only available with molecular genetic techniques.

There is another syndrome with a constellation of abnormalities associated with a predisposition to develop Wilms' tumour. Children noted at birth to have aniridia (absence of the iris of the eye) when this arises spontaneously, are known to have a 50% risk of developing Wilms' tumour (Fig 5). These anomalies are associated with deletion of part of the short arm of chromosome 11 (11p13). Examination of the chromosomes of one of our patients with banding techniques were initially reported as normal, but subsequent analysis has shown a tiny interstitial deletion in the 11p13 band, which is at the limit of resolution for even the most experienced cytogeneticists (Fig 6). Over the last few years it has been possible to localise a number of other genes in the vicinity of this Wilms'/aniridia locus, such as the genes for catalase, lactic dehydrogenase, beta globin, insulin, the Harvey ras oncogene, and the gene for parathyroid hormone. By mapping the genes that are present and absent in patients who have the deletion associated with Wilms' tumour, it has been possible to define to within very narrow limits, the area where the tumour gene itself resides (20) and it can only be a short time before the gene is identified and sequenced as was recently done for the retinoblastoma gene (21).

An awareness of this genetic mutation in patients with the aniridia-Wilms' syndrome has led us and many other groups to look in detail at the short arm of chromosome 11 in other patients with Wilms' tumour, and many of them have been found to have similar abnormalities. The technique used involves selecting a probe for a gene that is known to be in close proximity to the affected locus, such as one of the globin genes. After cutting the DNA with restriction enzymes, one looks for restriction fragments which show heterozygosity, that is the fragment containing the gene probe attaches to two fragments that migrate at different speeds on electrophoresis showing up as two bands on an autoradiograph. In patients whose lymphocytes show that their constitutional genotype is heterozygous for a probe, there is often loss of heterozygosity in the tumour tissue, where they have become homozygous (Fig 2). There are a number of different ways in which this can occur; among the commonest is simple loss of one chromosome 11, or loss followed by reduplication of the other chromosome.

Our present understanding of how this may result in tumour formation is as follows:-

(1) The patient may inherit a germ line abnormality of one chromosome 11, as in the patients born with aniridia.

(2) There may be a somatic mutation involving chromosome 11 in a clone of kidney cells.

(3) In either case a second event follows: this can be loss of the normal chromosome 11, with or without duplication of part of the defective chromosome. The end result is loss of heterozygosity, or homozygosity for the abnormal gene.

**Figure 6**

11p13 deletion in Wilms/Aniridia syndrome. (Courtesy of Regional Cytogentics Unit, Southmead Hospital.)

What this tells us is that the gene concerned must have some form of suppressor function since it is loss of the gene that results in tumour formation. Genes of this type as found in Wilms' tumour and Retinoblastoma have therefore been called antioncogenes.

### INTERCELLULAR COMMUNICATION

There are other examples of the importance of how cells talk to one another and of the consequences of keeping bad company. Teratomas, which have the capacity to develop 'normal' tissues of almost any type are among the most interesting tumours to study.

There is a characteristic form of teratoma that arises in the sacrococcygeal region in infants. If resected during the first few weeks of life these tumours are almost always benign, and yet if they are left in place or overlooked because they are of a small size until later, the majority become malignant and metastasise to other organs. Work is in hand in the CLIC Research Laboratories on cell cultures of these tissues though these tumours are very difficult to deal with in human cell cultures. Fortunately there is an excellent animal model in the mouse available for study. The importance of the company cells keep is well expressed in a classic experiment which uses stem cells from a mouse teratocarcinoma grown in vitro as a cell line (22). Under various conditions using different chemicals, these cells can be programmed to differentiate: and yet, the same cells put back into a mouse are tumourigenic and have therefore retained their capacity to be tumour cells. If the same cells are injected into a mouse blastocyst, which is then implanted back into the uterus, they become incorporated into the foetus and those mice when adult can be shown to be chimaeric, that is, some of their normal cells are derived from the abnormal tumour cells; they are now behaving normally because they are in good company. The contrary experiment also works: if you take normal cells from a mouse embryo at an early stage and inject them into another mouse, they will form a tumour or teratocarcinoma instead of a normal mouse embryo

This phenomenon is not restricted to teratoma cells. In a singularly important experiment reported recently by Leo Sachs and his colleagues from Israel, myeloid leukaemia cells, when injected into mice embryos were shown to undergo differentiation and to become part of the normal haematopoetic tissue of the adult mice (23). It is therefore possible to persuade some malignant cells back into a differentiative mode instead of a proliferative mode, a factor which is clearly of key importance when we come to think about the therapeutic possibilities available to us

### ONCOGENES ARE DERIVED FROM NORMAL GROWTH GENES

What is the evidence that proto-oncogenes participate in the normal development of the embryo? By plotting the expression of these genes during prenatal and postnatal development, it has been possible to establish characteristic patterns in different tissues, which show that they are in fact intimately involved in normal cellular proliferation and differentiation in the embryo and that they are under very careful control (24). Injection of such genes into the cells of an early embryo allows us to investigate their role in more detail. One of the first things to become clear from such experiments has been the finding that abnormal expression of a number of genes, e.g. c-myc, may result in characteristic malformations which are then inherited in subsequent generations (25). Similar experiments with the c-fos oncogene have also resulted in malformations of bones of a characteristic pattern (26).

### A CRISIS OF SELF-IDENTITY?

There is one further system concerned with how cells talk to one another that is important in this story. This is the vital system which detects when foreign material is present in the host and is able to mount an immune response to reject it. Without this system our bodies would be prey to every bacteria, virus and fungus in existence. The system was first defined in humans in terms of transplant immunology, where it is known as the HLA system (Human Leukocyte Antigen), but it is better known scientifically as the major histocompatibility complex (MHC). There are two major classes of MHC proteins which work with different classes of T lymphocytes to enable them to identify cells that have been infected with foreign antigens and to mount an immune response against them (27). Some of these MHC proteins 'mimic' viral proteins and then infections may result in auto-immune disorders. It should come as no surprise to learn that these MHC proteins may also be related to the development of malformations and malignancies.

In one of the commoner childhood tumours called Neuroblastoma, there is solid evidence for the involvement of the N-myc oncogene, usually located on chromosome 2. When this oncogene is amplified, as it commonly is in neuroblasoma, the excess DNA is often found in a characteristic form that is visible microscopically as 'double minutes' and as the tumour progresses, double minutes are incorporated somewhere in the genome as an 'homogeneously staining region' (HSR) which on analysis can be shown to be made up of multiple copies of N-myc DNA. There is a clear-cut relationship between the number of copies of the N-myc oncogene and the stage of progression of the Neuroblastoma

(28). Almost all the tumours diagnosed in an early stage of the disease have only one copy of the N-myc gene, whereas those with extensive disease have multiple copies. In the very small sub-section of patients with disseminated Neuroblastoma known to have a potential for spontaneous regression (Stage IV S), there is usually only one copy of the N-myc gene. An important clue as to how expression of this gene may correlate with the stage of the malignancy and with the ultimate survival of patients can be seen in experiments where extra copies of the N-myc gene have been transfected into a Neuroblastoma cell line. The presence of extra copies of N-myc resulted in down modulation and lack of expression of the MHC antigens on the cell surface (29). The contrary is malignant cell line so that the cells express the MHC proteins to a greater degree than before, this abolishes the cells capacity to metastasise, presumably because the body is now able to recognise them and deal with them (30).

Recently, Andrew Shaw in the CLIC Research Laboratories has been looking at this relationship in Wilms' tumour. Gillian Borthwick, also on a CLIC research grant has already investigated the expression of MHC antigens in Wilms' tumour and found that the malignant component of the tumour always lacks expression of these proteins in comparison with the normal stromal elements of the tumour. Andrew Shaw has now investigated the relationship between the expression of N-myc in Wilms' tumour cells and the expression of the MHC class 1 antigens. The expression of N-myc is much higher in Wilms' tumour cells than it is in normal kidney or lymphocyes from the same patient. Where N-myc is over expressed, the expression of the MHC class 1 antigens appears to be down modulated (31). It is clearly important to establish whether or not the expression of N-myc and of the MHC antigens in metastases is the same or different from that in the parent tumour: because of the excellent prognosis for this tumour on treatment, we are stil awaiting a suitable patient to study that phenomenon.

### A RATIONALE FOR COMPLEXITY

It is tempting when studying disorders such as malformations and malignancies to wonder whether a simpler cellular control system might not break down rather less often! The complexity of regulation is however something that has evolved over millions of years. We can certainly trace back many of the 'oncogenes' to simple forms of life such as bacteria and yeasts, so we presume that the laws of natural selection have provided us with a pretty fool-proof system which has got steadily better as the millenia have proceeded.

A good example of the need for complexity is seen in the requirement for the fetus to have a diversity of antibodies, capable of controlling the multiple infectious agents to which it is likely to be exposed after birth. If we look at how an antibody molecule is made, we can see that it involves the combination of a number of different genes. For the heavy chain, any one of a number of variable genes combines with one of a number of junctional genes joined with one of a number of diversity genes, and then finally with a constant gene before the messenger RNA is translated into the protein which makes up that particular chain. Calculation of the number of possible end products that could result reveals a total several thousand possibilities. When combined with a light chain to make a functional antibody, the number of possibilities rises to several million, before we even start to consider possible single point mutations in any one of these genes (32). The capacity for such diversity is obviously of crucial importance in the survival of the individual.

### FUTURE PROSPECTS

Finally I want to suggest a few examples of how molecular genetics should bring about fundamental changes in the practice of medicine within the next few years.

One of the most exciting areas of research is going to be into the identification of the benefits that accrue to the heterozygotes for many of the genes which in homozygous form result in the kind of rare and distressing diseases we have been discussing. The example of haemoglobinopathies is well known: the reason the genes for sickle cell anaemia and the Thalassaemias are so common in some parts of the world is because the heterozygotes are resistant to Malaria. Our understanding of that process has been of substantial benefit in many other areas. What about disorders such as Tay–Sach's Disease, which is such a scourge to some populations of Ashkenazi Jews? A map of the percentage of heterozygotes known to be present in various Jewish communities in Europe provides persuasive evidence that the gene frequency is highest in those areas with the worst experience of tuberculosis, suggesting that heterozygotes for this gene may be inherently resistant to tuberculosis (33). Identification of the gene and how it confers resistance to tuberculosis could be of major importance on a worldwide scale.

The simple ways in which genes can be modulated is also a fertile field of enquiry. The switch over from foetal to adult-type haemoglobin in the foetus has already been mentioned. In infants born to diabetic mothers, that switch is delayed, by mechanisms presently unclear (34). Understanding how to control the switch from one set of globin genes to another could again be of enormous benefit, for example in dealing with some forms of Thalassaemia.

The inheritance of diabetes is known to be related to genes of the MHC complex. There is preferential transmission of some alleles particularly from fathers, though also to a lesser degree from mothers. This clearly suggests a pre-natal advantage to the embryos within that genotype (35). What is it about that gene that confers this pre-natal advantage to the embryo? This surely might be of fundamental importance in many areas of medicine, once we understand how it works.

How is medical treatment going to change in this brave new world? The capacity to identify genetic disorders early in the life of the fetus may enable us to provide an appropriate, corrective environment to enable that fetus to grow and develop normally and to avoid the deleterious consequences of an 'abnormal' genotype (36). The abuse of the technology to allow the destruction of fetuses with an 'unwanted' genotype will be seen historically as an aberration of the first magnitude. Genes that have found their niche during evolution by conferring advantage on the heterozygote should be regarded as a vital and precious resource for the future of the human race. The challenge is surely to develop techniques to counterbalance the rare complications of inheriting the wrong 'dose' of such genes rather than to annihilate the unfortunate victim. Man's history of intolerance to the 'misfits' of our society is legendary, as is the record of the contribution of such misfits to our progress as a species.

It is already possible to synthesise proteins and enzymes with purposefully altered qualities that make them much more efficient in their desired effects than are the natural compounds (37).

It will be possible to take a clone of stem cells from an individual who has a genetic disorder, to alter the genetic component in vitro by replacing what is defective and to re-insert the stem cells into that individual, so partially correcting the deficit. This manoeuvre has already been carried our successfully in the Laboratory in a number of different experiments (38, 39).

The treatment of cancer is likely to undergo a revolutionary change from attempts to destroy malignant cells towards methods of rehabilitating them and rendering them innocuous. If we look at the oncogene story and how oncogenes produce tumour growth factors which make cells proliferate abnormally, it is possible to think of a number of different sectors on the communication pathway that we could interfere with to prevent tumour growth. Time and space do not permit us to look at the numerous examples of what has already been achieved in the Laboratory, but they are sufficient for us to be sure that entirely new methods for the treatment of cancer are also just 'around the corner'

The whole of medicine is going to reap the benefit of this new technology. It is sometimes difficult to believe that it is only five years since transgenic mice first hit the headlines and opened our eyes to what was to come. The general public is slowly being made aware of what this new science is all about and what it has to offer. Already recombinant DNA is manufacturing compounds which will alleviate many of the chronic diseases to which we are prone. With all the recent publicity about the tragedy of haemophiliacs who have developed AIDS because of the contamination of their supply of Factor VIII from human sources, it is public knowledge that human Factor VIII can now be manufactured by other species and will soon be commercially available in sufficient quantities to eradicate the risk of AIDS or other unsuspected infections in the future. The recent cloning of the Cystic Fibrosis gene has led to the hope that this dreadful disease will also soon and finally meet its match: as a bonus, we should also be able to define just what is the advantage to the heterozygotes for this autosomal recessive condition that makes the gene one of the most common in our society.

### CONCLUSION

I hope that despite the complexities of the subject, the general theme of growth and development has been apparent throughout this presentation. When Edward Long Fox qualified in 1857 and read the works of Rudolph Virchow and Charles Darwin, I wonder if he had any inkling of the revolutionary changes they were going to be responsible for in the next century. Yet I venture to suggest that the present changes in our understanding of disease at a molecular level will have an impact which will make the changes of the last century pale into insignificance.

It is perhaps fitting to end with what I shall call the 'Paediatrician's Creed' which will be just as true a century hence as it was in the time of Edward Long Fox, and is today — 'Infancy and childhood have implicit in them the beauty of growth and development and the anticipation of the future — the exquisite blend of nature and nurture, the genetic endowment and the impact of the environment' (40).

### REFERENCES

1 How to Nurse Sick Children. Published by Longman, Brown, Green and Longmans, London 1854.

2 LONG FOX, E. (1894) The Medical Man and the State, BMA Presidential Address.

3 WATSON, J. D., CRICK, F. H. C. (1953) A structure for deoxyribose nucleic acid, Nature **171**, 737.

4 REDDY, E. P., REYNOLDS, R. K., SANTOS, E., BARBACID, M. (1982) A point mutation is responsible for the acquisition of transforming properties by the T24 human bladder carcinoma oncogene, Nature **300**, 149.

5 SNYDER, S. (1985) The molecular basis of communication between cells, Scientific American **253**, 114–122.

6 WATERFIELD, M.D., SCRACE, G. T., WHITTLE, N., STROOBANT, P., JOHNSSON, A., WASTESON, A., WESTERMARK, B., HELDIN, C-H., HUANG, J. S., DEUEL, T. F. (1983) Platelet-derived growth factor is structurally related to the putative transforming protein p28 of simian sarcoma virus, Nature **304**, 35.

7 DOWNWARD, J., YARDEN, Y., MAYES, E., SCRACE, G., TOTTY, N., STOCKWELL, P., ULLRICH, A., SCHLESSINGER, J., WATERFIELD, M. D. (1984) Close similarity of epidermal growth factor receptor and v-erb-B oncogene protein sequences, Nature **807**, 521.

8 WEISS, R. A., MARSHALL, C. J. (1984) Oncogenes, *The Lancet*, Vol II, **1138**.

9 Gene amplification in malignancy. 1987 *The Lancet*, Vol I, **839**.

10 VAN BENEDEN, E. (1883) Recherches sur la maladie de l'ouef et la fecundation et la division cellulaire. *Arch. Biol.* **4**, 265–640.

11 BOVERI, T. (1914) Zur Frage der Entstelung maligner Tumoren. *Jena*.

12 KRIVIT, W., GOOD, R. A. (1956) Simultaneous occurrence of leukaemia and mongolism. *Am. J. Dis. Child.* **91**, 213.

13 BEDDIS, I., MOTT, M. G., BULLIMORE, J. (1983) Nasopharyngeal Rhabdomyosarcoma and Gorlin's Naevoid Basal Cell Carcinoma Syndrome. *Medical and Paediatric Oncology* **11**(3), 178–9.

14 TAYLOR, A. M., METCALFE, J. A., OXFORD, J. M. *et al.* (1976) Is chromatid-type damage in ataxia telangiectasia after irradiation at G a consequence of defective repair? *Nature* **260**, 441–443.

15 KERSEY, J., SPECTOR, B. D., GOOD, R. A. (1973) Primary immunodeficiency diseases and cancer: The Immunodeficiency Cancer Registry. *Int. J Cancer* **12**, 333–347

16 BOLANDE, R. P. (1976) Neoplasia of early life and its relationship to teratogenesis. In Perspectives in pediatric pathology, Year Book. *Med. Pub. Inc.* **3**, 145–184.

17 BADER, J. L., MILLER, R. W., MEADOWS, A. T. *et al.* (1980) Trilateral Retinoblastoma. *The Lancet.* **2**, 582.

18 GILBERT, F. (1986) Retinoblastoma and Cancer Genetics, NEJM, 1248–1250.

19 TURLEAU, C., de GROUCHY, J., CHAVIN-COLIN, F., MARTELLI, H., VOYER, M., CHARLAS, R. (1984) Trisomy 11p15 and Beckwith–Wiedemann syndrome. A report of two cases. *Human Genetics* **67**, 219–221.

20 KOUFOS, A., HANSEN, M. F., LAMPKIN, B. C., WORKMAN, M. L., COPELAND, N. G., JENKIN, N. A., CAVENEE, W. K. (1984) Loss of alleles at loci on human chromosome 11 during genesis of Wilms' tumour. *Nature,* **309**, 170–172.

21 LEE, W. W., BOOKSTEIN, R., HONG, F., YOUNG, L-J., SHEW, J-Y., LEE, Y-H. P. (1987) Human retinoblastoma susceptibility gene: cloning, identification, and sequence. *Science* **235**, 1394–1399.

22 DONY, C., KESSEL, M., GRUSS, P. (1985) Post-transcriptional control of myc and p53 expression during differentiation of the embryonal carcinoma cell line F9. *Nature* **317**, 636–639.

23 GOOTWINE, E., WEBB, C. G., SACHS, L. (1982) Participation of myeloid leukaemic cells injected into embryos in haematopoietic differentiation in adult mice. *Nature* **299**, 63–64.

24 MULLER, R., SLAMON, D. J., TREMBLAY, J. M., CLINE, M. J., VERMA, I. M. (1982) Differential expression of cellular oncogenes during pre- and postnatal development of the mouse. *Nature* **299**, 640–644.

25 WOYCHIK, R. P., STEWARD, T. A., DAVIS, L. G., D'EUSTACHIO, P., LEDER, P. (1985) An inherited limb deformity created by insertional mutagenesis in a transgenic mouse. *Nature* **318** 36–40.

26 RUTHER, U., GARBER, C., KOMITOWSKI, D., MULLER, R., WAGNER E. F. (1987) Deregulated c-fos expression interferes with normal bone development in transgenic mice. *Nature* **325**, 412–416.

27 HARELL, R. A., ALLEN, H. *et al.* (1986) Molecular Biology of the H-2 histocompatibility complex. *Science* **233**, 437–443.

28 SEEGER, R. C., BRODEUR, G. M., SATHER, H. *et al.* (1985) Association of multiple copies of the n-myc oncogene with rapid progression of neuroblastoma. *NEJM* **313**, 1111–1116.

29 BERNARDS, R., DESSALIN, S. K., WEINBERG, R. A. (1986) N-myc Amplification causes down-modulation of MHC class 1 antigen expression in neuroblastoma. *Cell* **47**, 667–674.

30 WALLICH, R., BULBUC, N., HAMMERLING, G. J., KATZAV, S., SEGAL, S., FIELDMAN, M. (1985) Abrogation of metastatic properties of tumour cells by de novo expression of H-2K antigens following H-2 gene transfection. *Nature* **315**, 301–305.

31 SHAW, A. P. W., POIRIER, V., TYLER, S., MOTT, M., BERRY, J., MAITLAND, N. J. (1988) Expression of the N-myc oncogene in Wilms' tumour and related tissues. *Oncogene* **3**, 143–149.

32 ROSENFIELD, I., ZIFF, E., VAN LOON, B. (1983) DNA for beginners, *Writers and Readers Publ. Inc.* London.

33 ROTTER, J. I., DIAMOND, J. M. (1987) What maintains the frequencies of human genetic diseases? *Nature*. **329**, 289–290.

*Continued on page 14.*

**Mutation, malformation and malignancy.**

*Continued from page 10.*

34 PERRINE, S. P., GREENE, M. F., FALLER, D. V. (1985) Delay in the fetal globin switch in infants of diabetic mothers. *NEJM* **312**, 334–338.

35 VADHEIM, C. M., ROTTER, J. I., MACLAREN, N. K., RILEY, W. J., ANDERSON, C. E. (1986) Preferential transmission of diabetic alleles within the HLA gene complex. *NEJM* **315**, 1314–1318.

36 ROSENBLATT, D. S., COOPER, B. A., SCHMUTZ, S. M., ZALENSKI, W. A., CASEY, R. E. (1985) Prenatal vitamin $B_{12}$ Therapy of a fetus with methylcobalamin deficiency (cobalamin E disease). *The Lancet*, 1127–130.

37 ROSENBERG, S., BARR, P. J., NAJARIAN, R. C., HALLEWELL, R. A. (1984) Synthesis in yeast of a functional oxidation-resistant mutant of human 1-antitrypsin. *Nature* **312**, 77–80

38 Le MEUR M, GERLINGER, P. BENOIST, C., MATHIS, D. (1985) Correcting an immune-response deficiency by creating $E_a$ gene transgenic mice, *Nature* **316**, 38–42.

39 WILLIAMS, D. A., LEMISCKA, I. R., NATHAN, D. G., MULLIGAN, R. C. (1984) Introduction of new genetic material into pluripotent haematopoietic stem cells of the mouse. *Nature* **310**, 476–480.

40 ZIAI, M. (1984) Textbook of Pediatrics. 3rd Edition, Little, Brown & Co, Boston.

## Figures and Tables

**Figure 1 f1:**
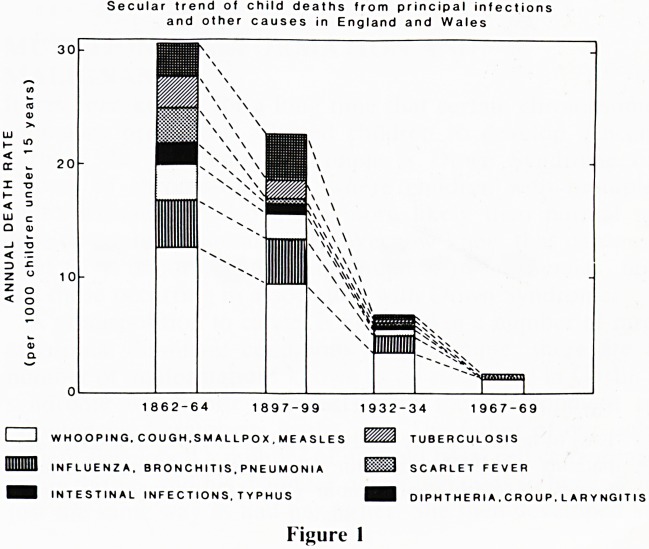


**Figure 2 f2:**
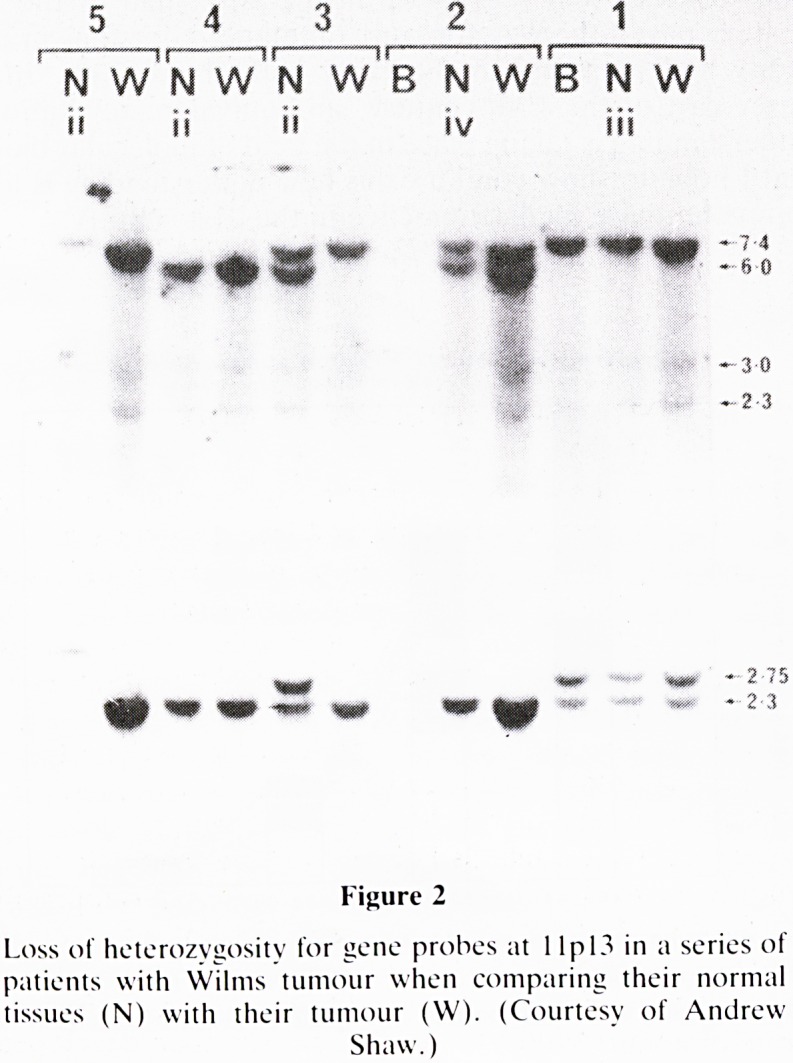


**Figure 3 f3:**
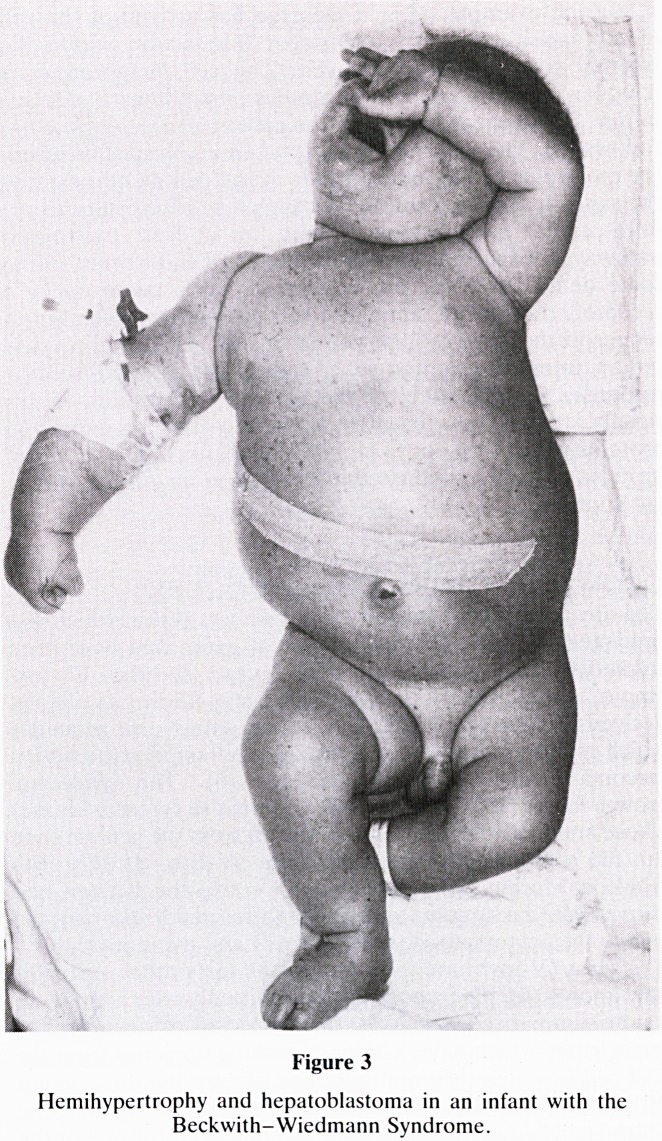


**Figure 4 f4:**
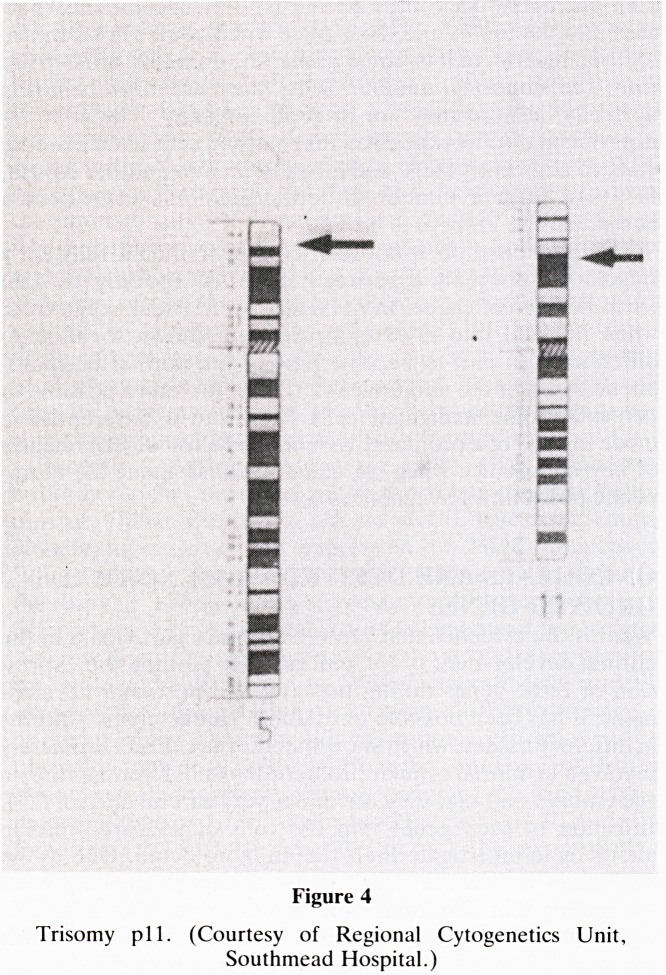


**Figure 5 f5:**
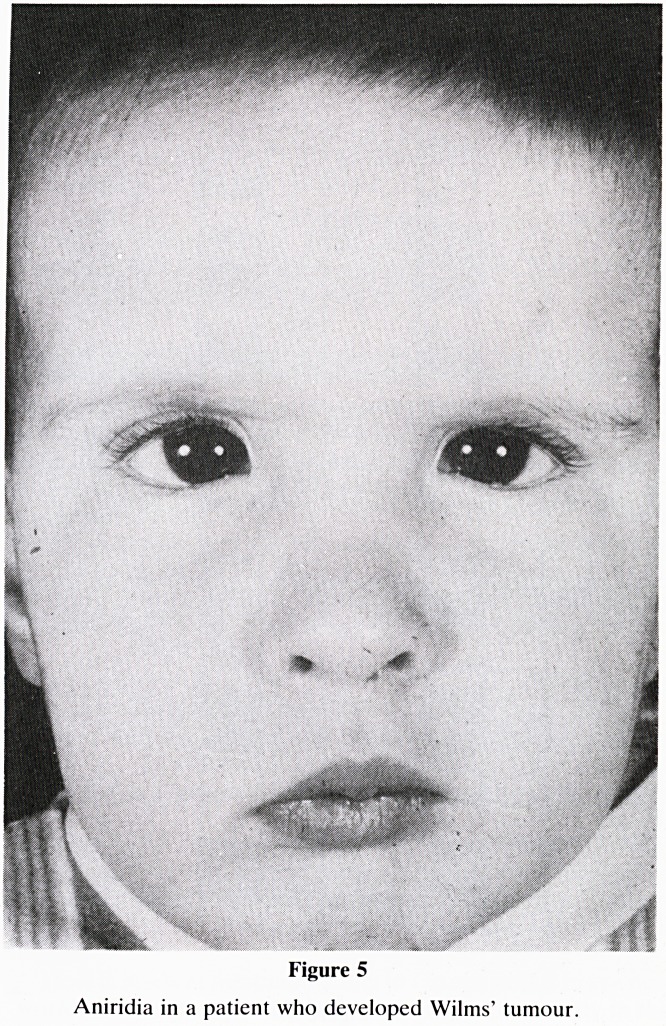


**Figure 6 f6:**